# Seasonal changes in free 25-(OH)D and vitamin D metabolite ratios and their relationship with psychophysical stress markers in male professional football players

**DOI:** 10.3389/fphys.2023.1258678

**Published:** 2023-10-16

**Authors:** Anna Książek, Aleksandra Zagrodna, Giovanni Lombardi, Małgorzata Słowińska-Lisowska

**Affiliations:** ^1^ Department of Biological and Medical Basis of Sport, Faculty of Physical Education and Sports, Wroclaw University of Health and Sport Sciences, Wroclaw, Poland; ^2^ Laboratory of Experimental Biochemistry and Molecular Biology, I.R.C.C.S. Istituto Ortopedico Galeazzi, Milan, Italy; ^3^ Department of Athletics, Strength and Conditioning, Poznań University of Physical Education, Poznań, Poland

**Keywords:** vitamin D, 24, 25-(OH)_2_D_3_, VMR, training load, athletes, seasonal variation

## Abstract

**Introduction:** Novel markers of vitamin D status are currently being investigated, including free 25-(OH)D (25-(OH)D_F_) and the vitamin D metabolite ratio (24,25-(OH)_2_D_3_:25-(OH)D_3_; VMR). The VMR may provide additional functional information on vitamin D metabolism in athletes. Therefore, the main objective of the current study was to evaluate 25-(OH)D_F_, bioavailable 25-(OH)D (25-(OH)D_B_), VMR, and psychophysical stress markers during different training periods over a half-season. The second aim was to assess the association between vitamin D binding protein (VDBP), total and free 25-(OH)D, VMRs, and psychophysical stress markers in professional football players. Moreover, we examined the relationship between 25-(OH)D_3_ and vitamin D metabolites (24,25-(OH)_2_D_3_, 3-epi-25-(OH)D_3_) to determine if training loads in different training periods influenced the vitamin D metabolome.

**Methods:** Twenty professional football players were tested at six different time points across half a year (V1—June; V2—July; V3—August; V4—October; V5—December; V6—January).

**Results:** Analyses indicated a significant seasonal rhythm for VDBP, and total 25-(OH)D (25-(OH)D_T_), 25-(OH)D_B_, 24,25-(OH)_2_D_3_, 3-epi-25-(OH)D_3_, 25-(OH)D_3_:24,25-(OH)_2_D_3_, and 24,25-(OH)_2_D_3_:25-(OH)D_3_ VMRs throughout the training period. No correlation was detected between 25-(OH)D_T_, 25-(OH)D_B_, 25-(OH)D_F_, vitamin D metabolites, VMRs, VDBP, and ferritin, liver enzymes (aspartate transaminase [AST] and alanine transaminase [ALT]), creatine kinase (CK), cortisol, testosterone, and testosterone-to-cortisol ratio (T/C) in each period (V1-V6). However, there was a strong statistically significant correlation between 25-(OH)D_3_ and 24,25-(OH)D_3_ in each training period.

**Conclusion:** In conclusion, a seasonal rhythm was present for VDBP, 25-(OH)D_T_, 25-(OH)D_B_, vitamin D metabolites (24,25-(OH)_2_D_3_, 3-epi-25-(OH)D_3_), and VMRs (25-(OH)D_3_:24,25-(OH)_2_D_3_, 25-(OH)D_3_:3-epi-25-(OH)D_3_). However, no rhythm was detected for 25-(OH)D_F_ and markers of psychophysical stress (ferritin, liver enzymes, CK, testosterone, cortisol, and T/C ratio). Moreover, the relationships between free and total 25-(OH)D with psychophysical stress markers did not demonstrate the superiority of free over total measurements. Furthermore, training loads in different training periods did not affect resting vitamin D metabolite concentrations in football players.

## 1 Introduction

Vitamin D is an important compound related to many aspects of athletic performance and recovery, with the most studied functions of vitamin D concerning bone (Herrmann et al., 2017) and skeletal muscle health (Dzik and Kaczor, 2019). Furthermore, vitamin D plays a vital role in modulating the functions of many other tissues that are important in a sports context, including those impacting immune (Martens et al., 2020) and cardiac function (Messa et al., 2014). Vitamin D status is a common topic in sports science due to the high prevalence of vitamin D insufficiency in athletes (Farrokhyar et al., 2015; Harju et al., 2022), which can negatively influence musculoskeletal function, power, force production, and recovery from fractures (Owens et al., 2018; Ribbans et al., 2021). All of these factors are pivotal for athletes as they may have impact on sport performance and general health.

In most athlete trials, serum 25-(OH)D_T_ level is a biomarker of vitamin D status due to its relatively long half-life. Consistent with the free hormone hypothesis (Chun et al., 2014), only the non-bounded fraction (the free fraction, 25-(OH)D_F_) can enter cells and exert its biological effects (Bikle, 2021). Some studies have shown that some vitamin D functions may be closely related to its free fraction than to VDBP bound 25-(OH)D_T_ concentrations (Powe et al., 2011; Shieh et al., 2018). VDBP levels and activities influence vitamin D bioavailability, altering the balance between free and bound vitamin D fractions (Bouillon et al., 2019). In such circumstances, 25-(OH)D_T_ concentration estimations may be misleading.

Some studies suggest (Cashman et al., 2015; Ginsberg et al., 2020) that evaluating the molar ratio of 24,25-(OH)_2_:25-(OH)D may be a better index of vitamin D insufficiency in healthy men (Alonso et al., 2022) as it is not affected by race (Berg et al., 2015). Recent evidence demonstrates that VMR strongly associates with higher bone mineral density (Ginsberg et al., 2018) and parathyroid hormone (PTH) (Bosworth et al., 2012) than 25-(OH)D_T_. Therefore, VMR may provide additional functional information on vitamin D metabolism in athletes.

The vitamin D receptor (VDR) is expressed in skeletal muscle and has essential roles in maintaining mitochondrial function and recovery (Latham et al., 2021). Latest evidence indicates that vitamin D signaling contributes to muscle regeneration. In animal models, VDR protein expression associates closely with 25-(OH)D serum concentration (Srikuea et al., 2020). The VDR and vitamin D-activating enzyme CYP27B1 are expressed at a low level in homeostatic skeletal muscle *in vitro* and *in vivo*, evidenced by immunocytochemical and immunohistochemical visualization and immunoblotting in C2C12 myoblasts and whole mouse muscle (Srikuea et al., 2012; Srikuea et al., 2020; Latham et al., 2021). Vitamin D play role in muscle regeneration supported by rapidly raised Pax7 and VDR protein expression in skeletal muscle to take action on the repair response after an acute bout of damaging high-intensity physical effort (Puangthong et al., 2021), demonstrating that the myogenic repair and vitamin D systems are both rapidly and contemporaneously initiated after skeletal muscle damage (Latham et al., 2021).

There are several studies on seasonal 25-(OH)D_T_ concentration changes in athletes (Lombardi et al., 2017; Vitale et al., 2018; Krzywański et al., 2020) though there are none on seasonal changes in 25-(OH)D_F_ and VMR variations. Moreover, fewer studies considered the different training periods in conjunction with vitamin D metabolites and VMRs. The main aim of the current study was to assess 25-(OH)D_F_, VMRs, (25-(OH)D_3_:24,25-(OH)_2_D_3_, 25-(OH)D_3_:3-epi-25-(OH)D_3_, 24,25-(OH)_2_D_3_: 25-(OH)D_3_), and psychophysical stress markers (ferritin, liver enzymes, creatine kinase [CK], testosterone, and cortisol) during different periods of the training season. The second aim was to examine the association between VDBP, total, bioavailable and free 25-(OH)D, VMRs, and psychophysical stress markers in professional football players. In addition, we assessed whether training loads in different training periods influenced the vitamin D metabolome. Therefore, we examined the relationship between 25-(OH)D_3_ and vitamin D metabolites (24,25-(OH)_2_D_3_, 3-epi-25-(OH)D_3_) in this cohort.

## 2 Materials and methods

### 2.1 Study design, participants, and blood draws

Forty-two football players were recruited from one club competing in the highest male football Polish league, the “Ekstraklasa,” providing a total of 180 records. After initial screening, the study included 20 participants with a mean age of 26.9 ± 4.7 years. Athletes with injuries, those not present at more than two blood draws, and participants who used calcium (Ca) or vitamin D supplementation were excluded. However, sporadic vitamin D intake was permitted. The competitors who participated in the study were active in all training periods and had similar athletic performance levels, career duration, and training loads.

Blood draws followed the first round of the Polish “PKO BP Ekstraklasa” from June 2021 to January 2022. Sample collections were performed in June - V1 (before the pre-season), July—V2 (after the pre-season), August - V3 (during the competitive season), October - V4 (during the competitive season), December—V5 (after the competitive season), and January - V6 (after off-season). The team trained regularly and played at latitudes between 50° and 54°N. Table 1 details the content of each of the training periods, considering different training sessions and durations.

**TABLE 1:** Training content for each of the training periods.

Day	Period	V1		V2		V3		V4		V5		V6	
		Before the pre-season		After the pre-season		During the competitive season		During the competitive season		After the competitive season		After the off-season	
		Duration		Duration		Duration		Duration		Duration		Duration	
Monday	M	60′	ITS	90′	CT		rest	70′	CT/TEC	95′	CT/TEC	45′	ITS
	A		Rest	80′	CT/TEC		rest		rest		rest		rest
Tuesday	M	45′	ITS	80′	CT	90′	CT/TEC	80′	CT	95′	CT/TEC	60′	ITS
	A		Rest	80′	TAC	80′	TAC	80′	TAC		rest		rest
Wednesday	M		Rest		rest		rest	60′	TAC		rest		rest
	A	60′	ITS	100′	CT/TEC	65′	CT/TAC		rest		rest	60′	ITS
Thursday	M		Rest	70′	CT/TEC	115′	CT/TEC	60′	CT/TEC	50′	CT/TEC		rest
	A	60′	ITS	60′	TAC		rest		rest		rest	90′	ITS
Friday	M		Rest	60′	CT	50′	TEC	60′	CT/TEC	80′	CT/TEC		rest
	A	45′	ITS	90′	TEC		rest		rest		rest	45′	ITS
Saturday	M		Rest	90′	FG	50′	TAC		rest	40′	TAC		Rest
	A		Rest		rest		rest	90′	LG		rest		Rest
Sunday	M		Rest		rest	90′	LG		rest		rest		Rest
	A		Rest		rest		rest		rest	90′	LG		Rest

M, morning; A, afternoon; ITS, individual training session; CT, conditioning training (interval training, strength training, stamina training, speed training); TAC, tactical training; TEC, technical training; FG, friendly game; LG, league game.

### 2.2 Biochemical analyses

Blood samples were collected into plain tubes containing a clot activator (Vacutest, Kima, Italy), stored at room temperature for 1 hour, then centrifuged at 1300 *g* for 10 minutes at 22 °C. Serum aliquots were stored at −80°C until assayed.

As previously described (Książek et al., 2022), total serum Ca was determined by colorimetric assay using the Konelab 60 system (bioMérieux, Marcy-l’Etoile, France). Albumin was assayed on a Siemens Dimension Xpand Plus clinical chemistry system (Siemens, Munich, Germany).

Albumin-adjusted Ca (ACa) was calculated using the formula:

$$\text{ACa }\left(\text{mg}/\text{dL}\right)=\text{Ca }\left(\text{mg}/\text{dL}\right)+\left[\left(4-\text{albumin }\left(g/\text{dL}\right)\right)*0.8\right]$$

Intact PTH (iPTH) was determined in serum by an electrochemiluminescence assay (ECLIA) on an Elecsys analyzer (Roche, Basel, Switzerland). The intra- and inter-assay coefficients of variation (CVs) were 4.5% and 4.8%, respectively, and the limit of detection was at 1.20 pg .mL^-1^ (0.127 pmol .L^-1^).

Plasma CK activity was evaluated using diagnostic kits for the Konelap 60 kinetic enzyme analyzer (bioMérieux, Marcy-l’Etoile, France). The CK detection limit for the kits was 6 U/l, with an intra-assay CV of 1.85%.

Serum ferritin and cortisol levels were measured by ECLIA on the Cobas e601 analyzer (Roche, Mannheim, Germany). Intra-assay and inter-assay CVs for ferritin and cortisol were 2.5% and 8.1%, and 5.4% and 10.1%, respectively. Serum total testosterone was measured by ECLIA on the Cobas e411 analyzer (Roche, Mannheim, Germany) and had intra-assay and inter-assay CVs of 4.7% and 8.4%, respectively.

The testosterone-to-cortisol ratio (T/C) was calculated as a surrogate marker of overtraining and psychophysical stress.

Aspartate transaminase (AST) and alanine transaminase (ALT) levels were measured by enzymatic assay on the Alinity m analyzer (Abbott Laboratories, IL, USA). The AST intra- and inter-assay CVs were 0.7% and 1.0%, respectively, and the limit of detection was 3 U/L. The ALT intra- and inter-assay CVs were 0.9% and 1.5%, respectively, and the limit of detection was 2 U/L.

### 2.3 Vitamin D metabolite levels

Vitamin D metabolite levels 25-(OH)D_3_, 24,25-(OH)D_2_, 25-(OH)_2_D_3_, and 3-epi-25-(OH)D_3_), and 25-(OH)D_3_-to-24,25-(OH)_2_D_3_, 25-(OH)D_3_-to-3-epi-25-(OH)D_3_, and 24,25-(OH)_2_D_3_-to-25-(OH)D_3_ ratios were quantitatively determined by liquid chromatography coupled with tandem mass spectrometry (LC-MS/MS).

Vitamin D metabolite standards (25-(OH)D_3_, 3-epi-25-(OH)D_3_, 25-(OH)D_2_, 24,25-(OH)_2_D_3_) and isotope internal standards (25-(OH)D_3_-^13^C_5_, 24,25-(OH)_3_D3-^2^H_6_, 25-(OH)D_2_-^2^H_3_, 3-epi-25-(OH)D_3_-^2^H_3_) hereafter called IS mixture were all obtained from IsoSciences (Ambler, PA, United States). In order to prepare calibration standards, vitamin D-free serum was purchased from Golden West Diagnostics (Temecula, CA, United States). The following reagents were used for the preparation of mobile phases or samples: acetonitrile (ACN), methanol (MeOH), water (H_2_O), ethyl acetate, and formic acid (FA). The reagents listed above were obtained from VWR (Pennsylvania, United States). A derivatization reaction was carried out using 4-(4′-dimethylaminophenyl)-1,2,4-triazoline-3,5-dione (DAPTAD) synthesized by Masdiag Laboratory (Warsaw, Poland). A chromatographic separation was performed using a Zorbax Eclipse XDB-C18 (1.8µm, 80Å, 100 × 4.6 mm, Agilent Nacalai Tesque, Santa Clara, United States).

The LC-MS analyses were conducted using a Nexera XR HPLC system (Shimadzu, Kyoto, Japan) with an Eksigent autosampler (Sciex, Framingham, MA, United States) coupled with a QTRAP^®^ 5500 mass spectrometer (Sciex, Framingham, MA, United States). Analyses were performed in multiple reaction monitoring (MRM) in positive ionization using electrospray source (ESI). These ion source parameters were used: Temperature (TEM) 550°C, Curtain Gas (CUR) 25 psi, IonSpray Voltage (IS) 4000 V, Ion Source Gas 1 (GS1) 40 psi, Ion Source Gas 2 (GS2) 70 psi. H_2_O (phase A) and ACN (phase B), both containing 0.1% formic acid, were used as mobile phases, with a flow rate of 0.6 mL/min. Gradient elution was performed as follows: 0 min - 50% B, 2.5 min - 78% B, 3.2 min - 98% B, 4.5 min - 98% B, 4.6 min - 50% B, 5.5 min - 50% B. Total run time was 5.5 min. The column oven temperature was 45°C.

### 2.4 Free 25-(OH)D levels from vitamin D binding protein analysis

VDBP concentration was measured using a commercially available enzyme-linked immunosorbent assay (ELISA) (R&D Systems, MN, United States). The intra-assay CV ranges between 5% and 7%, and the inter-assay CV ranges from 5% to 8%.

Free vitamin D (25-(OH)D_F_) levels were estimated using a published mathematical method (Bikle et al., 1986), with the affinity binding constants for 25-(OH)D_T_ with albumin and VDBP being 6 × 10^5^ M^−1^ and 7 × 10^8^ M^−1^, respectively.

$$Free\;25-\left(OH\right)D=\frac{total\;25-\left(OH\right)D}{1+\left(6\;x\;10^{5}x\;albumin\right)+\left(7\;x\;10^{8}\;x\;VDBP\right)}$$

The levels of bioavailable 25-(OH)D (25-(OH)D_B_) were calculated using equations adapted from Powe et al. (Powe et al., 2011)

$$Bioavailable\;25-\left(OH\right)D=\left(1+\left(6\;x\;10^{5}\right)\;x\;albumin\right)\;x\;Free\;25-\left(OH\right)D$$

The concentration of 25-(OH)D_F_ and 25-(OH)D_B_ were derived from the respective total (sum of D_3_ + D_2_) values.

### 2.5 Statistical analysis

The sample size was estimated using the G power 3.1.9.2 calculator (http://www.gpower.hhu.de/). Descriptive statistics were presented using mean and standard deviation. The Shapiro-Wilk test assessed data normality, and Levene’s test examined homogeneity of variance. Differences between seasons (summer—V1+V2+V3 vs. winter - V4+V5+V6) were assessed using comparison of mixed models with and without seasonal effect, with players id as random effect. VDBP, vitamin D metabolites (25-(OH)D_T_, 25-(OH)D_F_, 24,25-(OH)_2_D_3_, 3-epi-25-(OH)D_3_), VMRs, and psychophysical stress markers were processed with single and population mean cosinor tests to evaluate the presence of a seasonal rhythm. Seasonal rhythm analysis was performed based on the midline estimate statistic rhythm (MESOR) (defined as the rhythm-adjusted mean value) using the cosinor-fitting equation, y = MESOR + Amplitude x cos (Frequency (x) + acrophase), where acrophase is the difference (time) between the MESOR and peak value in the cosine curve. Multiple regression analysis assessed the association between VDBP, 25-(OH)D_T_, 25-(OH)D_F_, VMR, and psychophysical stress markers, while Pearson’s correlation coefficient determined the association between 25-(OH)D_3_ and vitamin D metabolites. All analyses employed R for Windows, version 4.3.1 (R Foundation for Statistical Computing, Vienna, Austria) (Team, 2016). The significance threshold was *p* < 0.05.

## 3 Results

The data analysis included results from 20 football players. Changes in biochemical parameter levels during the training periods are shown in Table 2. Significant changes in 25-(OH)D_T_, 25-(OH)D_B_, 24,25-(OH)_2_D_3_, 3-epi-25-(OH)D_3_, 25-(OH)D_3_:24,25-(OH)_2_D_3_, and 24,25-(OH)_2_D_3_:25-(OH)D_3_ VMR concentrations were demonstrated between the measurements recorded throughout the training period (Table 2). There were no differences in 25-(OH)D_3_:3-epi-25-(OH)D_3_ VMR, ferritin, AST, ALT, CK, hs-CRP, testosterone, cortisol and T/C ratio between training periods in studied athletes.

**TABLE 2:** Changes in the levels of the biochemical parameters of football players (n = 20) during the training periods (V1-V6).

	V1	V2	V3	V4	V5	V6	p
VDBP (μmol/L)	2.65 ± 0.74	2.66 ± 0.87	2.97 ± 0.87	2.28 ± 0.94	2.13 ± 0.89	1.42 ± 1.06	
25-(OH)D_T_ (ng/mL)	42.0 ± 6.2	45.0 ± 5.9	48.6 ± 7.3	41.8 ± 5.8	37.9 ± 6.1	33.7 ± 4.5	p_V1-V3_ < 0.001, p_V1-V6_ = 0.002, p_V2-V3_ = 0.017, p_V2-V4_ = 0.022, p_V2-V5_ = 0.003, p_V2-V6_< 0.001, p_V3-V4_< 0.001, p_V3-V5_< 0.001, p_V3-V6_< 0.001, p_V4-V6_ = 0.002
25-(OH)D_F_ (pg/mL)	20.1 ± 5.9	21.7 ± 5.6	21.2 ± 5.1	20.3 ± 4.6	19.5 ± 5.3	18.6 ± 3.9	
25-(OH)D_B_ (ng/mL)	7.8 ± 2.3	8.7 ± 2.4	8.6 ± 1.9	8.0 ± 1.9	7.7 ± 2.0	5.8 ± 3.3	p_V1-V5_ < 0.001, p_V2-V5_ < 0.001, p_V3-V5_ < 0.001, p_V4-V5_ < 0.001
24,25-(OH)_2_D_3_ (ng/mL)	3.8 ± 0.9	4.3 ± 0.8	4.6 ± 1.0	3.8 ± 0.8	3.2 ± 0.7	2.8 ± 0.7	p_V1-V2_ = 0.031, p_V1-V3_ = 0.003, p_V1-V6_ = 0.017, p_V2-V3_ = 0.028, p_V1-V5_ < 0.001, p_V1-V6_ < 0.001, p_V3-V4_ = 0.016, p_V3-V5_ < 0.001, p_V3-V6_ < 0.001, p_V4-V5_ = 0.02, p_V4-V6_ = 0.01
3-epi-25-(OH)D_3_ (ng/mL)	2.0 ± 0.8	2.1 ± 0.5	2.3 ± 0.9	1.8 ± 0.5	1.6 ± 0.7	1.3 ± 0.4	p_V3-V6_ = 0.038
25-(OH)D_3_:24,25-(OH)_2_D_3_ VMR	11.4 ± 1.7	10.6 ± 1.1	10.5 ± 1.2	11.0 ± 1.4	11.7 ± 1.8	12.1 ± 2.4	p_V1-V2_ = 0.021, p_V1-V3_ = 0.005, p_V3-V5_ = 0.02
24,25-(OH)_2_D_3_:25-(OH)D_3_ x100 VMR	8.94 ± 1.43	9.52 ± 0.95	9.65 ± 1.12	9.22 ± 1.18	8.73 ± 1.32	7.31 ± 3.40	p_V1-V2_ = 0.011, p_V1-V3_ = 0.015, p_V2-V5_ = 0.022, p_V3-V5_ = 0.017
25-(OH)D_3_:3-epi-25-(OH)D_3_ VMR	23.2 ± 6.7	21.8 ± 4.3	22.6 ± 6.7	23.9 ± 6.6	26.3 ± 8.4	27.2 ± 7.7	
Ferritin (µg/dL)	107.9 ± 66.4	107.1 ± 68.0	105.0 ± 75.3	104.7 ± 31.3	90.3 ± 30.3	109.9 ± 81.4	
AST [U/L]	29.8 ± 11.9	29.6 ± 5.3	28.0 ± 7.5	25.9 ± 5.3	24.9 ± 5.1	26.9 ± 9.4	
ALT [U/L]	25.4 ± 8.1	24.2 ± 4.8	25.0 ± 6.5	21.7 ± 4.2	23.8 ± 7.0	21.4 ± 5.2	
CK [UI/L]	439.4 ± 241.6	539.6 ± 771.3	380.7 ± 246.4	372.2 ± 186.8	480.4 ± 537.4	243.9 ± 97.8	
hs-CRP [mg/L]	3.08 ± 0.17	3.14 ± 0.18	3.21 ± 0.13	3.14 ± 0.21	3.24 ± 0.13	3.16 ± 0.2	
Testosterone [nmol/L]	24.3 ± 6.0	22.8 ± 5.2	23.0 ± 5.6	23.2 ± 6.7	24.3 ± 6.0	22.3 ± 4.6	
Cortisol [nmol/L]	458.5 ± 71.2	464.4 ± 80.2	457.7 ± 64.4	471.9 ± 69.3	492.1 ± 72.8	456.1 ± 90.9	
T/C ratio	0.05 ± 0.02	0.05 ± 0.02	0.05 ± 0.02	0.05 ± 0.02	0.05 ± 0.01	0.04 ± 0.02	

VDBP, vitamin D binding protein; AST (asparte), ALT (alanine)—liver enzymes; CK, creatin kinase; T/C–testosterone to cortisol ratio.

We also investigated the differences between seasons (summer vs. winter) in VDBP, vitamin D metabolites, VMR and psychophysical stress markers in football players. Table 3 shows changes in the levels of biochemical parameter between studied periods (summer (V1-V3) and winter (V4-V6)). In summer VDBP (*p* = 0.013), 25-(OH)D_T_ (*p* < 0.001), 25-(OH)D_B_ (*p* = 0.006), 24,25-(OH)_2_D_3_, 3-epi-25-(OH)D_3_ (*p* < 0.001) and 24,25-(OH)_2_D_3_:25-(OH)D_3_ (*p* = 0.011) were significantly higher than in winter period. We also observed that during summer (which covered preparatory and competitive training periods) AST (*p* = 0.027) and ALT (*p* = 0.018) were significantly higher than during winter. In winter 25-(OH)D_3_:24,25-(OH)_2_D_3_ (*p* = 0.004) and 25-(OH)D_3_:3-epi-25-(OH)D_3_ VMR (*p* = 0.012) were significantly higher than in summer.

**TABLE 3:** Changes in the levels of the biochemical parameters of football players (n = 20) between summer and winter period.

	Summer (V1+V2+V3)	Winter (V4+V5+V6)	p
VDBP (μmol/L)	2.76 ± 0.15	1.95 ± 0.37	0.013
25-(OH)D_T_ (ng/mL)	45.25 ± 6.94	38.08 ± 6.34	<0.001
25-(OH)D_F_ (pg/mL)	21.00 ± 5.45	19.52 ± 4.61	0.12
25-(OH)D_B_ (ng/mL)	8.38 ± 2.20	7.20 ± 2.61	0.006
24,25-(OH)_2_D_3_ (ng/mL)	4.21 ± 0.93	3.32 ± 0.86	<0.001
3-epi-25-(OH)D_3_ (ng/mL)	2.15 ± 0.76	1.58 ± 0.58	<0.001
25-(OH)D_3_:24,25-(OH)_2_D_3_ VMR	10.85 ± 1.40	11.56 ± 1.86	0.004
24,25-(OH)_2_D_3_:25-(OH)D_3_ x100 VMR	9.37 ± 1.20	8.47 ± 2.24	0.011
25-(OH)D_3_:3-epi-25-(OH)D_3_VMR	22.53 ± 5.89	25.71 ± 7.53	0.012
Ferritin (µg/dL)	106.48 ± 68.58	100.20 ± 51.32	0.64
AST [U/L]	29.14 ± 8.53	25.84 ± 6.66	0.027
ALT [U/L]	24.88 ± 6.50	22.38 ± 5.59	0.018
CK [UI/L]	452.98 ± 478.17	360.22 ± 330.55	0.23
hs-CRP [mg/L]	3.15 ± 0.17	3.18 ± 0.19	0.32
Testosterone [nmol/L]	23.37 ± 5.55	23.34 ± 5.85	0.68
Cortisol [nmol/L]	460.25 ± 70.82	474.56 ± 77.08	0.24
T/C ratio	0.05 ± 0.02	0.05 ± 0.02	0.51

VDBP, vitamin D binding protein; AST (asparte), ALT (alanine)—liver enzymes; CK, creatin kinase; T/C–testosterone to cortisol ratio.

### 3.1 Mean percent 3-epi-25-(OH)D_3_ of 25-(OH)D

3-epi-25-(OH)D_3_ was detectable in each training period in the entire study cohort. The mean percent 3-epi-25-(OH)D_3_ of 25-(OH)D was 4.9%, ranging from 1.3% to 4.3% (V1), 4.7% ranging from 1.4% to 3.0% (V2), 4.8% ranging from 1.4% to 4.2% (V3), 4.4% ranging from 1.1% to 3.0% (V4), 4.1% ranging from 0.9% to 3.5% (V5), 3.6% ranging from 0.8% to 2.0% (V6).

### 3.2 Seasonal rhythmicity

**FIGURE 1:**
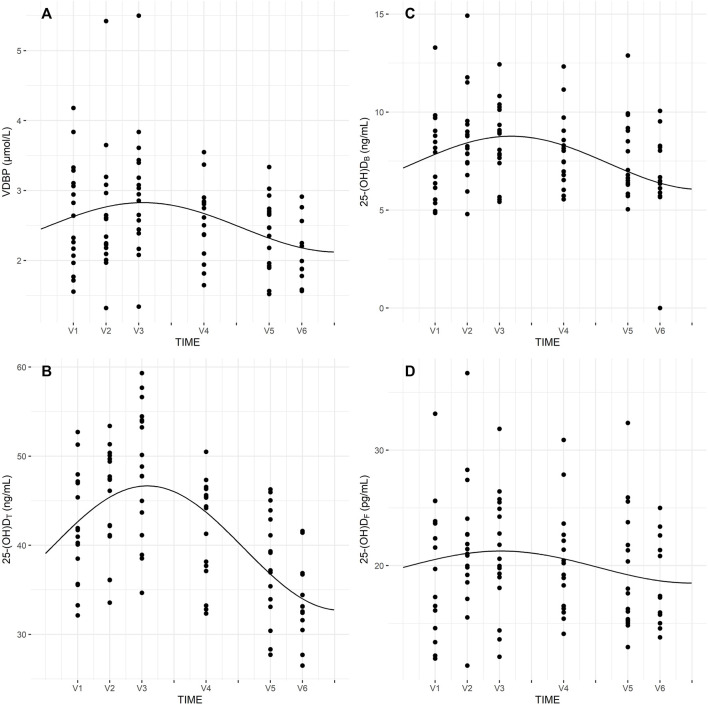
Seasonal rhythm of VDBP **(A)**, 25-(OH)D_T_ **(B)**, 25-(OH)D_B_ **(C)**, 25-(OH)D_F_ **(D)**.

**FIGURE 2:**
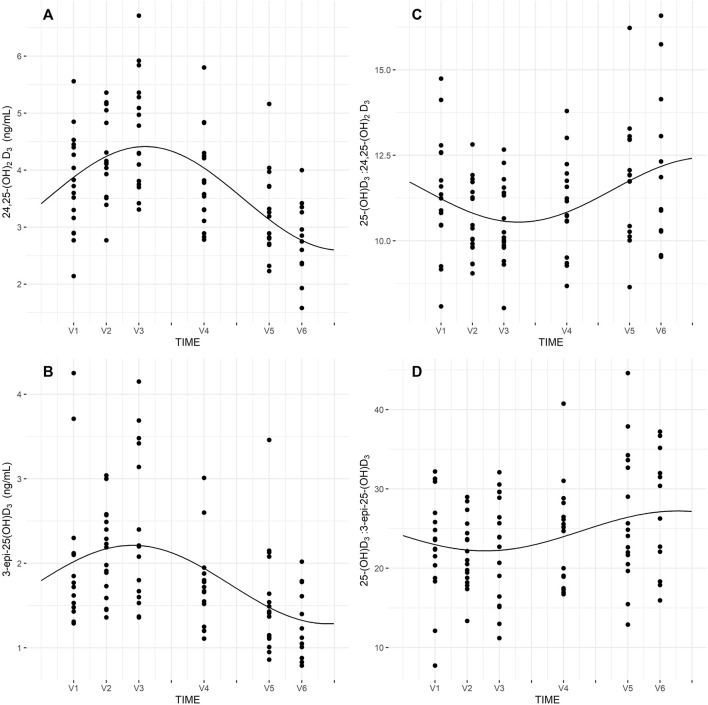
Seasona rhythm of 24,25-(OH)_2_D_3_ **(A)**, 3-epi-25-(OH)D_3_ **(B)**, 25-(OH)D_3_:24,25-(OH)_2_D_3_ **(C)**, 25-(OH)D_3_:3-epi-25-(OH)D_3_ **(D)**.

Seasonal rhythm analysis was performed to evaluate training period-related fluctuation in VDBP, vitamin D metabolites (25-(OH)D_T_, 25-(OH)D_F_, 24,25-(OH)_2_D_3_, 3-epi-25-(OH)D_3_), VMRs, and psychophysical stress markers. Cosinor curves (Figure 1, Figure 2) showed significant seasonal rhythm for VDBP (*p* = 0.003), 25-(OH)D_T_, 25-(OH)D_B_, 24,25-(OH)_2_D_3_, 3-epi-25-(OH)D_3_, 25-(OH)D_3_:24,25-(OH)_2_D_3_ VMR (*p* < 0.001), and 25-(OH)D_3_:3-epi-25-(OH)D_3_ VMR (*p* = 0.007). No significant rhythm was observed for 25-(OH)D_F_ and all psychophysical stress markers (ferritin, liver enzymes, CK, testosterone, cortisol, and T/C ratio). Table 4 shows the rhythmometric parameters of vitamin D metabolites and ratios.

**TABLE 4:** Rhythmometric analysis of VDBP, vitamin D metabolites and ratios of football players.

	PR [%]	*p*-value	MESOR [mean and 95% cl]	Amplitude [mean and 95% cl]	Acrophase [mean and 95% cl]
VDBP (μmol/L)	94	0.003	2.47 [2.30–2.65]	0.35 [0.09–0.62]	−1.50 [-2.19 to −0.81]
25-(OH)D_T_ [ng/mL]	35.3	<0.001	39.70 [38.20–41.19]	6.97 [4.66–9.28]	−1.48 [−1.79 to −1.18]
25-(OH)D_B_ [ng/mL]	11.6	<0.001	7.41 [6.85–7.96]	1.36 [0.46–2.26]	−1.38 [−1.93 to −0.82]
25-(OH)D_F_ [pg/mL]	32	0.082	19.87 [18.61–21.12]	1.39 [-0.48–3.26]	−1.55 [-2.85 to −0.24]
24,25-(OH)_2_D_3_ [ng/mL]	32.5	<0.001	3.50 [3.30–3.71]	0.91 [0.59–1.23]	−1.47 [−1.78 to −1.16]
3-epi-25-(OH)D_3_ [ng/mL]	19.6	<0.001	1.75 [1.59–1.91]	0.46 [0.24–0.69]	1.47 [0.93–2.01]
25(OH)D_3_:24,25(OH)_2_D_3_	10.7	0.001	11.49 [11.11–11.88]	0.95 [0.30–1.59]	−1.33 [−1.85 to −0.81]
25(OH)D_3_:3-epi-25(OH)D_3_	7.5	0.007	24.70 [23.07–26.34]	2.51 [0.41–4.62]	1.34 [0.27–2.40]

PR: percentage of rhythm. MESOR: Midline Estimating Statistic of Rhythm. Amplitude: half the difference between the highest and the lowest points of the cosine function best fitting the data. Acrophase indicates the time in which the highest values occur.

### 3.3 VDBP, 25-(OH)D_F_, vitamin D metabolites, VMRs and psychophysical stress markers

Multiple regression analysis was performed to analyze the association between VDBP, 25-(OH)D_T_, 25-(OH)D_B_, 25-(OH)D_F_, vitamin D metabolites, VMRs, and psychophysical stress markers. However, no correlation was detected between VDBP, 25-(OH)D_T_, 25-(OH)D_B_, 25-(OH)D_F_, 24,25-(OH)_2_D_3_, 3-epi-25-(OH)D_3_, 25-(OH)D_3_:24,25-(OH)_2_D_3_, 25-(OH)D_3_:24,25-(OH)_2_D_3_, 25-(OH)D_3_:3-epi-25-(OH)D_3_, ferritin, liver enzymes (AST, ALT), CK, cortisol, testosterone, and T/C ratio in each period (V1-V6).

### 3.4 25-(OH)D_3_, 24,25-(OH)_2_D_3_ and 3-epi-25-(OH)D_3_

Pearson’s correlation coefficient was calculated to define the association between 25-(OH)D_3_ and vitamin D metabolites during different training periods. Table 5 shows the relationship between 25-(OH)D_3_ and vitamin D metabolites. There was a strong statistically significant association between 25-(OH)D_3_ and 24,25-(OH)D_3_ in each training period (V1-V4: *p* < 0.001; V5: *p* < 0.01; V6: *p* < 0.01). The association between 25-(OH)D_3_ and 3-epi-25(OH)D_3_ was observed only after the pre-season period (V2: *p* < 0.01) (Table 5).

**TABLE 5:** Correlations between 25-(OH)D_3_ and vitamin D metabolites in each training period.

	25(OH)D_3_ (ng/mL)					
Vitamin D metabolites						
	V1	V2	V3	V4	V5	V6
	R					
24,25(OH)_2_D_3_ (ng/mL)	0.75^b^	0.85^b^	0.83^b^	0.81^b^	0.69^a^	0.74^a^
3-epi-25(OH)D_3_ (ng/mL)	−0.02	0.63^a^	0.46	0.43	0.48	0.28

^a^ *p* < 0.01.

^b^ *p* < 0.001.

## 4 Discussion

This is the first study to examine seasonal changes in vitamin D metabolites, VMRs, and markers of psychophysical stress in male football players.

We found significant seasonal rhythms for VDBP, 25-(OH)D_T_, and 25-(OH)D_B_. Similarly, Vitale el al. (Vitale et al., 2018). observed a significant circannual rhythm in 25-(OH)D_T_ concentrations in male and female professional skiers. The higher vitamin D concentrations appeared in July, with the rhythm-adjusted mean and amplitude comparable between the two groups. Lombardi et al. (Lombardi et al., 2017) evaluated vitamin D, CK, testosterone, and cortisol in three professional football teams to investigate circannual rhythms. The authors documented statistically significant circannual rhythms for 25-(OH)D_T_ cortisol, testosterone and T/C ratio. In contrast, the current study found no seasonal rhythm for these psychophysical stress markers.

Our results indicated significant seasonal rhythms in 24,25-(OH)_2_D_3_, 3-epi-25-(OH)D_3_, 25-(OH)D_3_:24,25-(OH)_2_D_3_, and 25-(OH)D_3_:3-epi-25-(OH)D_3_ ratios. Tang et al. (Tang et al., 2019) conducted research on 940 young and healthy United Kingdom army recruits and found circannual rhythms for all vitamin D metabolites (25-(OH)D, 24,25-(OH)_2_D) and VMRs (25-(OH)D:24,25-(OH)_2_D, 1,25-(OH)_2_D:24,25-(OH)_2_D), except for 1,25-(OH)_2_D, when fitted to cosinor curves. Our cohort of professional football players showed similar trends. Indeed, 3-epi-25-(OH)D_3_ and 24,25-(OH)_2_D_3_ had a propensity to fluctuate with 25-(OH)D_T_, with changes between summer and winter periods. Furthermore, 25-(OH)D_3_:24,25-(OH)_2_D_3_ and 25-(OH)D_3_:3-epi-25-(OH)D_3_ VMRs were less susceptible to seasonal fluctuation. It should be noted that sunlight exposure is one of the major factor, which influences on vitamin D metabolites levels, especially in countries situated at latitude above 40°. Therefore, the results of this study show that higher concentration of vitamin D metabolites (25-(OH)D_T_, 25-(OH)D_B,_ 25-(OH)D_F_, 24,25-(OH)_2_D_3_, 3-epi-25-(OH)D_3_) occurred in summer time (July, August) compare to fall or winter months. Other factors, which may affecting on vitamin D metabolite levels is dietary intake of vitamin D or use of supplements.

The primary aim of athletes training is to supply stimulation that disrupts homeostasis to bring about adaptive responses that enhance physical performance. Therefore, maximizing the training stimulus is a key rule of athletic training. On the other hand, the ability to recover fast is crucial so that competitors can perform at high intensities more frequently. Human skeletal muscle counters to training stimuli and/or tissue damage through remodeling (Dahlquist et al., 2015). Some recent studies suggest that vitamin D might play an important role in skeletal muscle repair and remodeling (Owens et al., 2018), and others have reported on the relationship between 25-(OH)D_T_ muscle damage biomarkers, and overtraining symptoms. The results of these studies found no relationship between 25-(OH)D_T_ and ferritin (Jastrzebska et al., 2017) or CK (Lombardi et al., 2017; Ferrari et al., 2020) in athletes from different sports disciplines. Based on the free hormone hypothesis, some vitamin D functions may be closely linked to its free fraction than total serum 25-(OH)D concentrations (Powe et al., 2011; Shieh et al., 2016). Hence, we examined whether 25-(OH)D_F_ was associated better with psychophysical stress markers than 25-(OH)D_T_. However, we documented no significant association of 25-(OH)D fractions (total and free) with psychophysical stress markers in football players in each training period.

Testosterone is the principal male sex hormone and stimulates anabolic metabolism, causing an increase in muscle and skeletal system volume, strength, and endurance. Moreover, testosterone facilitates muscular adaptations to exercise and improves their recovery ability. Male reproductive physiology is influenced by 25-(OH)D (Crewther et al., 2011), with VDRs and vitamin D metabolizing enzymes expressed in Leydig cells (Jensen, 2012; Boisen et al., 2017), suggesting a direct role for vitamin D in steroidogenesis regulation. Based on this evidence, vitamin D may have a role in regulating testosterone levels. However, we found no correlation between 25-(OH)D_T_, 25-(OH)D_F_, and testosterone concentration in football players, which is in line with our previous study (Książek et al., 2021) in young, healthy men. Krzywański et al. (Krzywański et al., 2020) also evaluated the relationship between plasma 25-(OH)D_T_ and testosterone concentrations in professional track and field athletes. Similar to the current study, they found no significant correlation between 25-(OH)D_T_ and testosterone concentrations in male and female athletes, from strength or endurance disciplines, in any season. Crewther et al. (Crewther et al., 2020) assessed the interplay between 25-(OH)D_T_, cortisol, and testosterone and their effects on exercise performance in 88 male ice hockey players (<20 years), with no correlation evident between vitamin D and both hormones. Other studies also demonstrated no relationship between 25-(OH)D_T_ and cortisol and testosterone concentration in elite soccer players (Lombardi et al., 2017; Ferrari et al., 2020) and ice hockey players (Fitzgerald et al., 2018).

The effects of a single exercise on vitamin D (Sun et al., 2017; Dzik et al., 2022) and its metabolites (Mieszkowski et al., 2020) have been documented. However, there are no such data on the effects of varied training loads over different training periods on the vitamin D metabolome. Therefore, we explored the relationship between 25-(OH)D_3_, 24,25-(OH)_2_D_3_, and 3-epi-25-(OH)D_3_ in each training period, and found that 25-(OH)D_3_ correlated strongly with 24,25-(OH)_2_D_3_ in all periods. Although 24,25-(OH)_2_D and 3-epi-25-(OH)D are deemed to be biologically inactive metabolites, studies in animal models indicated that 24,25-(OH)_2_D exerts a pivotal role in maintaining bone integrity, function, and healing (Seo et al., 1997; Seo and Norman, 1997). Moreover, 3-epi-25-(OH)D_3_ levels are directly connected with the cardiovascular risk profile, and 3-epi-1α,25(OH)_2_D, a derivative of 3-epi-25-(OH)D, successfully decrease blood PTH without affecting Ca levels (Brown et al., 2005; Lutsey et al., 2015). The biological function of 24,25-(OH)_2_D and 3-epi-25-(OH)D is not well understood. Therefore, studying the effects of vitamin D metabolite actions on skeletal muscle function in athletes is vital.

This study had strengths and limitations. One of the study’s strengths is the homogeneity of the cohort and the athletes having similar training loads/volumes. In addition, vitamin D metabolites were measured using gold-standard methods. The main limitation of this study was that the data collection period covered only half of the year instead of the whole year. Also, a greater number of participants would have increased the power of statistical analysis. Nevertheless, football teams usually have no more than 25 players. Moreover, we assessed 25-(OH)D_F_ concentrations using a calculated method rather than directly measuring free vitamin D metabolite levels. Nonetheless, findings emerging from various studies indicate that VMRs, particularly the 24,25-(OH)_2_D-to-25-(OH)D ratio (i.e., the ratio indicating how much precursor could be converted into the bioactive form), may better represent the vitamin D status than 25-(OH)D alone since it considers the different metabolic fates. Specifically, the ratio of 24,25-(OH)_2_D:25-(OH)D should range between 4% and 12%, which reflects correct vitamin D status regardless of the absolute 25-(OH)D level (Alonso et al., 2022).

## 5 Conclusion

In conclusion, a seasonal rhythm was present for VDBP, 25-(OH)D_T_, 25-(OH)D_B_, vitamin D metabolites (24,25-(OH)_2_D_3_, 3-epi-25-(OH)D_3_), and VMRs (25-(OH)D_3_:24,25-(OH)_2_D_3_, 25-(OH)D_3_:3-epi-25-(OH)D_3_), though none was detected for 25-(OH)D_F_ or psychophysical stress markers (ferritin, liver enzymes, CK, testosterone, cortisol, and T/C ratio). Furthermore, no correlation was observed between total or free 25-(OH)D, VMRs, or psychophysical stress markers. As such, the association between free and total 25-(OH)D and psychophysical stress markers do not demonstrate the superiority of free measurements over total measurements. The results of the present study did not provide evidence that 25-(OH)D_T_ and 25-(OH)D_F_ influence testosterone concentration in football players during different training periods. Moreover, training loads in different training periods did not affect resting vitamin D metabolite concentrations.

## Data Availability

The raw data supporting the conclusion of this article will be made available by the authors, without undue reservation.
